# Genetic polymorphism analyses of 30 InDels in Chinese Xibe ethnic group and its population genetic differentiations with other groups

**DOI:** 10.1038/srep08260

**Published:** 2015-02-05

**Authors:** Hao-Tian Meng, Yu-Dang Zhang, Chun-Mei Shen, Guo-Lian Yuan, Chun-Hua Yang, Rui Jin, Jiang-Wei Yan, Hong-Dan Wang, Wen-Juan Liu, Hang Jing, Bo-Feng Zhu

**Affiliations:** 1 Research Center of Stomatology, Stomatological Hospital, Xi'an Jiaotong University, Xi'an. 710004, P. R. China; 2 Xi'an Jiaotong University Health Science Center, Xi'an 710061, P. R. China; 3 Blood Center of Shaanxi Province, Xi'an 710061, P. R. China; 4 College of Life Sciences, Shaanxi Normal University, Xi'an 710062, China; 5 Scientific Research and Experiment Center, The Second Affiliated Hospital, School of Medicine, Xi'an Jiaotong University, Xi'an 710004, P. R. China; 6 People's hospital of Arong Banner, Hulun Buir City, 162750. P. R. China; 7 The Second Affiliated Hospital of Xi'an Jiaotong University, Xi'an. 710004, P. R. China; 8 Key Laboratory of Genome Sciences, Beijing Institute of Genomics, Chinese Academy of Sciences, Beijing 100101, P. R. China; 9 Medical Genetic Institute of Henan Province, People's Hospital of Henan Province, Zhengzhou 450003, P. R. China

## Abstract

In the present study, we obtained population genetic data and forensic parameters of 30 InDel loci in Chinese Xibe ethnic group from northwestern China and studied the genetic relationships between the studied Xibe group and other reference groups. The observed heterozygosities ranged from 0.1704 at HLD118 locus to 0.5247 at HLD92 locus while the expected heterozygosities ranged from 0.1559 at HLD118 locus to 0.4997 at HLD101 locus. The cumulative power of exclusion and total probability of discrimination power in the studied group were 0.9867 and 0.9999999999902 for the 30 loci, respectively. Analyses of structure, PCA, interpopulation differentiations and phylogenetic tree revealed that the Xibe group had close genetic relationships with South Korean, Beijing Han and Guangdong Han groups. The results indicated that these 30 loci should only be used as a complement for autosomal STRs in paternity cases but could provide an acceptable level of discrimination in forensic identification cases in the studied Xibe group. Further studies should be conducted for better understanding of the Xibe genetic background.

At present, short tandem repeats (STRs) are widely used in forensic cases for paternity testing and individual identification in worldwide forensic DNA laboratories^1^,^2^,^3^,^4^. The universal use of STRs leads to the establishment of well constructed forensic DNA databases and development of simple typing methodologies. However, the STR-based genotyping in forensic applications has some limitations as follows, the relatively long amplicon size of STR would have negative influence in analyzing highly degraded DNA samples from crime cases^5^,^6^,^7^; artifacts, such as stutter peaks would add ambiguity to mixture analysis^8^,^9^; and the relatively high mutation rate of STR (approximately 10^−3^)^10^ would confound the kinship results. To overcome such limitations for STR loci, single nucleotide polymorphisms (SNPs) are considered as an alternative and supplementary markers^11^. SNPs have smaller amplicons than STRs; do not produce stutter peaks in typing profilers; and have a lower mutation rate (approximately 10^−8^)^12^,^13^. Nevertheless, SNP-based genotyping is usually complex, expensive and platform-dependent, hard to be conducted in common forensic laboratories^14^,^15^.

Insertion deletion polymorphisms (InDels) are recently gained the attention of forensic scientists. InDels as biallelic polymorphic markers that caused by the insertion or deletion of bases, combine the desirable characteristics of both SNPs and STRs. Similar to SNPs, InDels have small amplicons and relatively low mutation rates^16^. However, as length polymorphisms, InDels can be genotyped using capillary electrophoresis which is available in common forensic laboratories. InDels are the second abundant DNA polymorphisms after SNPs^17^. About 20% of the variations in human genome are InDels^18^. In 2002, Weber et al.^18^ reported 2000 biallelic human InDels and the population data in four groups (Europeans, Africans, Japaneses and Native Americans). Later in 2006, Mills et al.^19^ provided an initial map of InDels with more than 415,000 polymorphisms. Since then, InDels were used for a wide range of purposes such as study the biogeographic ancestry of human population^20^, use as genetic markers in natural populations^21^,^22^, and assess individual interethnic admixture and population substructure^23^. So far, one commercial InDel kit is available, i.e the Qiagen Investigator DIPplex kit (Qiagen, Hilden, Germany), and some population data obtained using this kit were published^22^,^24^,^25^,^26^. The allele frequencies of InDels are different among population groups in geographically separated areas. This makes InDels got the potential as ancestry informative markers^18^,^27^. However, at the same time, this also makes it necessary for us to assure the population indices before use InDels in new populations.

Xibe is one of the 56 ethnic groups officially recognized by the People's Republic of China. The Xibe group is widely distributed in the northern part of China from Xinjiang Uygur Autonomous Region in the west to Jilin and Liaoning provinces in the east (http://english.peopledaily.com.cn/102759/7567650.html). In the 6^th^ China population census in 2010, the population of Xibe is 190,481, ranking the 31^st^ in all the ethnic groups in China.

In this study, we used the mentioned Investigator DIPplex kit to obtain the population data of the studied Xibe ethnic group. To date, no genetic data of these 30 InDel loci was reported in Xibe group. The present study provided the population data and enriched the genetic informational resources of Chinese minority ethnic groups. The data were then used to calculate the forensic and population parameters and to make comparisons with other populations reported previously, providing information for the potential use of these loci in forensic cases and furthering the understanding of the genetic relationships between the Xibe group and other groups.

## Methods

### Sample preparation

Bloodstain samples were collected from 223 unrelated healthy Xibe individuals living in Ili, Xinjiang Uygur Autonomous Region, China. Before sample collection, all the participants signed the informed consent after given an explanation about this study. Volunteers in this study should have ancestors living in the region for more than three generations and have no common ancestry tracing back more than three generations. The study was conducted in accordance with the human and ethical research principles of Xi'an Jiaotong University Health Science Center, China and approved by the ethics committee of Xi'an Jiaotong University Health Science Center. The genomic DNA was extracted from bloodstain samples using the Chelex-100 method as described by Walsh et al.^28^.

### InDel typing

In this study, the commercially available InDel kit: Investigator DIPplex kit (Qiagen, Hilden, Germany) was used for InDel genotyping of 30 InDel loci. According to the manufacturer's protocol, we optimized the PCR volume to 25 μL, containing 5 μL Reaction Mix A, 5 μL Primer Mix and 0.6 μL Multi Taq2 DNA Polymerase, the template DNA and nuclease-free water. Amplification was carried out by a GeneAmp PCR System 9700 Thermal Cycler (Applied Biosystems, Foster City, CA, USA) under the following conditions: initial denaturation at 94°C for 4 min, followed by 30 cycles of 94°C for 30 s, 61°C for 2 min, 72°C for 75 s, and additional 60 min at 68°C. Electrophoresis was performed using the ABI PRISM 3130 Genetic Analyzer (Applied Biosystems, Foster City, CA, USA) under the conditions described in the manufacturer's recommendations using the denaturing polymer POP-4. Fragment sizing was supported using the BTO 550 (Qiagen, Hilden, Germany) as internal lane standard. The alleles were genotyped using the GeneMapper® ID software v3.2 (Applied Biosystems, Foster City, CA, USA). Control DNA 9948 was used as amplification positive control.

### Quality control

The study was conducted following ISFG recommendations on the analysis of the DNA polymorphisms as described by Schneider^29^.

### Statistical analyses

Allele frequencies and forensic efficiency parameters including observed (Ho) and expected (He) heterozygosity, Hardy-Weinberg equilibrium (HWE), polymorphic information content (PIC), power of exclusion (PE), discrimination power (DP) and typical paternity index (TPI) were calculated using the modified powerstat (version1.2) spreadsheet. *Fst* value was used to measure variance in allele frequencies among different populations. Population structure analysis was conducted using the structure program (version 2.2)^30^. Principal component analysis (PCA) based on allele frequencies was performed in MATLAB 2007a (MathWorks Inc., USA). Phylogenetic reconstruction was conducted utilizing the genetic distance and using phylogenetic analysis (DISPAN) program.

## Results and discussions

### Forensic parameter analysis

Allele frequencies and forensic statistical parameters of the 30 InDel loci were shown in Table 1. The Ho ranged from 0.1704 at HLD118 locus to 0.5247 at HLD92 locus while the He ranged from 0.1559 at HLD118 locus to 0.4997 at HLD101 locus. In the test of HWE, the genotype frequency distributions showed no significant deviations from expectations, with the lowest *p*-value of 0.0660 at HLD97 locus. PIC of all selected loci ranged from 0.1437 (HLD118) to 0.3749 (HLD101). The highest PE was found at HLD92 locus (0.2100), with the lowest found at HLD118 locus (0.0222). The values of DP were in the range of 0.2827 (HLD118)-0.6322 (HLD101). The highest, lowest and average TPI were 1.0519 (HLD92), 0.6027 (HLD118) and 0.8725, respectively. The cumulative power of exclusion (the probability to exclude the unrelated male from putative father using the full-loci data, indicating the forensic efficiency of the loci in paternity testing in the group) and combined discrimination power (the probability to distinguish two randomly chose individuals using the full-loci data, indicating the forensic efficiency of the loci in individual identification in the group) were 0.9867 and 0.9999999999902 for the DIPplex kit in the studied Xibe group, respectively. The value of cumulative power of exclusion was relatively low, which indicated that the DIPplex kit should only be used as a complement for autosomal STRs in paternity cases, such as in cases with mutations on autosomal STRs. Meanwhile, the value of combined discrimination power was high enough to give an acceptable level of discrimination in forensic identification cases.

### Clustering by structure analysis

**Figure 1:**
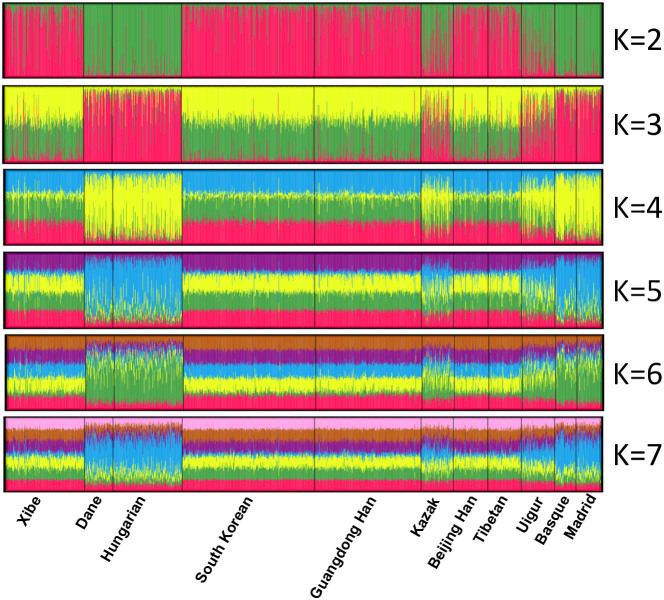
Clustering analysis by structure for the full-loci dataset assuming *K* = 2–7. Population names were labeled beneath.

We analyzed the population structures of the studied Xibe group and 10 referenced groups (South Korean^31^, (Madrid) Central Spanish, Basque (Northern Spanish)^32^, Hungarian^33^, Dane^34^, Beijing Han (Northern Han Chinese), Tibetan, Uigur, Kazak^22^ and Guangdong Han (Southern Han Chinese)^35^) by the structure program. The result was shown in Fig. 1. At *K* = 2, the clusters were anchored by Europe and Asia, and the 4 European groups and 5 Asian groups were constituted almost entirely by green and red component, respectively. At the same time, Kazak and Uigur group showed a mixed constitution of both green and red component. At *K* > 2, the 4 European groups and the 2 Eurasian groups (Kazak and Uigur groups) all had a mean component representing the European descent, while the 5 Asian groups had partial membership in K-1 clusters with similar membership proportions. According to the structure manual^30^, this indicated that the 5 groups showed similar membership proportions at the 30 loci. Moreover, as mentioned by Rosenberg et al., groups which had similar membership proportions in different clusters might reflect continuous gradations in allele frequencies across regions or admixture of neighboring groups^36^.

### Principal component analysis

**Figure 2:**
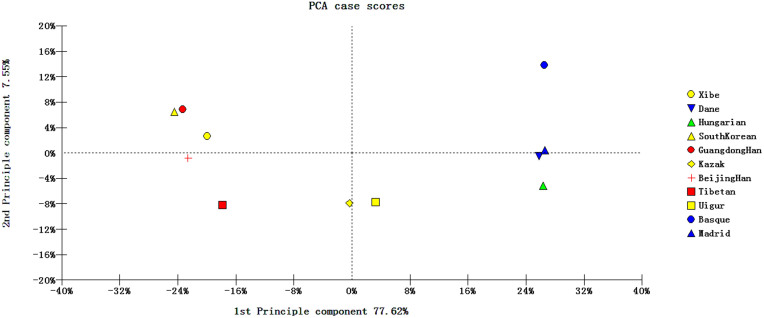
PCA based on 30 InDel loci of Xibe and 10 reference groups.

PCA was performed among the studied Xibe group and other 10 reference groups on the 30 InDel loci. The result was shown in Fig. 2. The first two principal components defined 85.17% of the total variance, with the first and second component accounted for 77.62% and 7.55%, respectively. According to the figure, the 4 European groups and 5 Asian groups distributed in the right and left part, respectively, with the 2 Eurasian groups (Kazak and Uigur groups) located between them, that is, in the middle of the plot. The studied Xibe group clustered in the upper left quadrant, close to Beijing Han, Guangdong Han and South Korean populations, indicating the close genetic relationships between the studied Xibe group and these groups.

### Interpopulation differentiation

The studied Xibe group was compared with previously published groups at the 30 InDel loci utilizing analysis of molecular variance method. The *Fst* and *p*-values were shown in Table 2. Statistically significant differences (*p* < 0.05) were observed between the studied Xibe group and South Korean group at 4 loci; Madrid group at 17 loci; Basque group at 17 loci; Hungarian group at 18 loci; Dane group at 16 loci; Beijing Han group at 2 loci; Tibetan group at 6 loci; Uigur group at 9 loci; Kazak group at 7 loci; Guangdong Han group at 6 loci. According to the results, East Asian (South Korean, Beijing Han and Guangdong Han groups) and Eurasian groups (Kazak and Uigur groups) had fewer differences (significant differences found at less than 10 loci) with the Xibe group than Europe groups (significant differences found at more than 15 loci), being consistent with the geographic distances between the studied Xibe group and these groups. Among the 30 loci, the highest ethnic diversity was obtained at 6 loci (HLD39, HLD58, HLD64, HLD84, HLD99 and HLD111) with significant differences found between the studied Xibe group and 7 other compared groups, followed by HLD81, HLD118 and HLD131 (6 groups). The lowest ethnic diversity was obtained at 4 loci (HLD70, HLD92, HLD101 and HLD124) with no significant difference found between the studied Xibe group and all other compared groups. The results showed that, ethnic diversity varied between different InDel loci. Some loci showed no significant difference even between groups from different continent, while others would have significant differences between groups with relatively close relationship. Hence, study of more InDel loci in more ethnic groups should be useful for screening InDel loci with different ethnic diversities for different purposes. And the genetic profile would also help us to gain a better understanding of the national evolutionary history.

### Phylogenetic analysis

**Figure 3:**
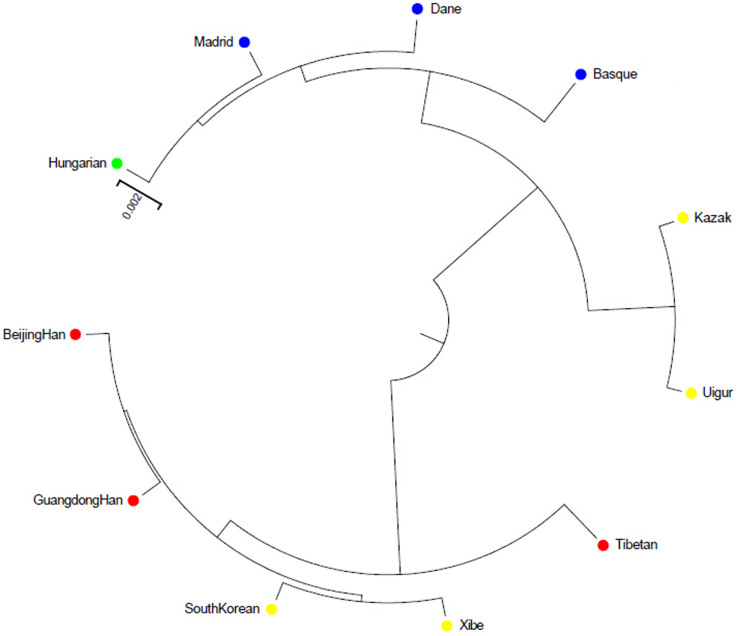
Phylogenic tree constructed by the unweighted pair-group method with arithmetic means based on the 30 InDel loci of the Xibe group and 10 reference groups.

Phylogenetic reconstruction was conducted to illustrate the genetic relationships between the studied Xibe group and the reference groups using the unweighted pair-group method with arithmetic means method (UPGMA). The UPGMA tree was shown in Fig. 3. The dendrogram showed 2 main clusters. The first cluster was composed of two branches: one included Hungarian, Madrid (Central Spanish), Dane, Basque Country (Northern Spanish); the other included Kazak and Uigur, respectively. The second cluster was composed of Beijing Han, Guangdong Han, South Korean, Xibe and Tibetan groups. The result was consistent with the above mentioned results of structure and PCA. The Xibe group was first clustered with the South Korean group, followed by Beijing Han and Guangdong Han group, then the Tibetan group. Zheng et al. constructed a phylogenetic tree based on 17 Y-STR loci (AmpFlSTR® Y-filer™ PCR Amplification kit, Applied Biosystems, Foster City, CA, USA) revealed that the Xibe group from Xinjiang was clustered with Chinese Korean group before clustered with Shandong Han population^37^. A study on mitochondrial DNA of Xibe group also reported an unrooted Neighbor-Joining tree indicating that Xibe group from Xinjiang had a close relationship with Chinese Korean group, Northern Han group and Southern Han group, and the relationship between Xibe and Korean group was closer than that between Xibe and the two Han groups^38^. Since the ancestors of Chinese Korean ethnic group migrated from the Korean peninsula from about the late 17th century, Chinese Korean ethnic group and South Korean group had the same ancestry origin^39^. Before the Qing government moved the Xibe ethnic group with people of some other ethnic minorities to Xinjiang to consolidate and reinforce the northwestern border defenses in the mid-18th century, they lived in the northeast China, where was also the residence of Chinese Korean group^39^. The close geographic distance may lead to intermarriage, and gene flow would be one of the reasons that cause the close relationship between Xibe and Korean group.

## Conclusions

In summary, the results indicated that these 30 loci should only be used as a complement for autosomal STRs in paternity cases but could provide an acceptable level of discrimination in forensic cases in the studied Xibe group. Analyses of structure, principal component analysis, interpopulation differentiations, and phylogenetic tree revealed the studied Xibe group had a close relationship with South Korea, Beijing Han and Guangdong Han groups. Further studies of comparison between Xibe group and more reference groups would be helpful for the better understanding of the Xibe genetic background.

## Author Contributions

H.M. and B.Z. wrote the main manuscript text, Y.Z., C.S., G.Y., C.Y., R.J., W.L. and H.W. did the data processing and the manuscript modification, J.Y. and H.J. prepared the figures. All authors reviewed the manuscript.

## Figures and Tables

**Table 1:** Allele frequency distribution and forensic statistical parameters of the 30 Indel loci in Chinese Xibe ethnic group (*n* = 223)

HLD	rs#	DIP−	DIP+	Ho	He	*p*	PIC	PE	DP	TPI
6	1610905	0.4574	0.5426	0.4843	0.4964	0.6938	0.3732	0.1741	0.6288	0.9696
39	17878444	0.8475	0.1525	0.2511	0.2584	0.7877	0.2250	0.0453	0.4150	0.6677
40	2307956	0.3767	0.6233	0.5022	0.4696	0.3444	0.3593	0.1895	0.5935	1.0045
45	2307959	0.3677	0.6323	0.4484	0.4650	0.5980	0.3569	0.1462	0.6118	0.9065
48	28369942	0.5919	0.4081	0.5022	0.4831	0.5895	0.3664	0.1895	0.6070	1.0045
56	2308292	0.4013	0.5987	0.4529	0.4805	0.3910	0.3651	0.1495	0.6258	0.9139
58	1610937	0.6996	0.3004	0.3946	0.4204	0.4197	0.3320	0.1107	0.5814	0.8259
64	1610935	0.2354	0.7646	0.3363	0.3600	0.4464	0.2952	0.0796	0.5267	0.7534
67	1305056	0.2848	0.7152	0.4350	0.4073	0.4168	0.3244	0.1367	0.5585	0.8849
70	2307652	0.3834	0.6166	0.4350	0.4728	0.2447	0.3610	0.1367	0.6240	0.8849
77	1611048	0.4843	0.5157	0.5022	0.4995	0.9616	0.3748	0.1895	0.6234	1.0045
81	17879936	0.1570	0.8430	0.2422	0.2646	0.4350	0.2296	0.0423	0.4188	0.6598
83	2308072	0.5942	0.4058	0.4798	0.4823	0.9161	0.3660	0.1704	0.6167	0.9612
84	3081400	0.1973	0.8027	0.2780	0.3168	0.2058	0.2666	0.0549	0.4788	0.6925
88	8190570	0.4215	0.5785	0.4574	0.4877	0.3485	0.3688	0.1529	0.6313	0.9215
92	17174476	0.5179	0.4821	0.5247	0.4994	0.4701	0.3747	0.2100	0.6111	1.0519
93	2307570	0.3789	0.6211	0.4978	0.4707	0.4364	0.3599	0.1855	0.5968	0.9955
97	17238892	0.6256	0.3744	0.4081	0.4685	0.0660	0.3587	0.1189	0.6268	0.8447
99	2308163	0.1570	0.8430	0.2242	0.2646	0.1653	0.2296	0.0367	0.4134	0.6445
101	2307433	0.5112	0.4888	0.4843	0.4997	0.6207	0.3749	0.1741	0.6322	0.9696
111	1305047	0.8767	0.1233	0.2287	0.2162	0.6638	0.1928	0.0381	0.3665	0.6483
114	2307581	0.6794	0.3206	0.4439	0.4357	0.8257	0.3408	0.1430	0.5840	0.8992
118	16438	0.0852	0.9148	0.1704	0.1559	0.5601	0.1437	0.0222	0.2827	0.6027
122	8178524	0.7265	0.2735	0.3767	0.3974	0.5091	0.3185	0.1004	0.5613	0.8022
124	6481	0.4126	0.5874	0.5112	0.4847	0.4476	0.3672	0.1975	0.6039	1.0229
125	16388	0.5919	0.4081	0.4753	0.4831	0.7915	0.3664	0.1668	0.6195	0.9530
128	2307924	0.6390	0.3610	0.4260	0.4614	0.2759	0.3549	0.1305	0.6151	0.8711
131	1611001	0.6861	0.3139	0.3946	0.4307	0.2636	0.3380	0.1107	0.5918	0.8259
133	2067235	0.5942	0.4058	0.4709	0.4823	0.7088	0.3660	0.1632	0.6206	0.9449
136	16363	0.4574	0.5426	0.5202	0.4964	0.4979	0.3732	0.2058	0.6107	1.0421

HLD, human locus deletion/insertion polymorphism; DIP−, frequency of short allele; DIP+, frequency of long allele; Ho, observed heterozygosity; He, expected heterozygosity; *p*, *p*-value for Hardy-Weinberg equilibrium; PIC, polymorphic information content; PE, power of exclusion; DP, discrimination power; TPI, typical paternity index.

**Table 2:** *Fst* and *p*-values of pairwise InDel loci between Chinese Xibe group and other groups at 30 InDel loci

HLD	South Korean		Madrid		Basque		Hungarian		Dane		Beijing Han		Tibetan		Uigur		Kazak		Guangdong Han	
	*Fst*	*p*	*Fst*	*p*	*Fst*	*p*	*Fst*	*p*	*Fst*	*p*	*Fst*	*p*	*Fst*	*p*	*Fst*	*p*	*Fst*	*p*	*Fst*	*p*
6	0.0225	**0.0110**	−0.0021	0.6026	−0.0053	1.0000	N	N	−0.0037	0.7994	0.0159	0.1124	0.0044	0.2692	−0.0038	0.9036	−0.0004	0.5484	0.0158	**0.0350**
39	0.0010	0.3780	0.1619	**0.0000**	0.0445	**0.0216**	0.1313	**0.0000**	0.0635	**0.0052**	−0.0030	0.7392	0.0310	**0.0410**	0.0697	**0.0026**	0.0139	0.1502	0.0137	**0.0434**
40	−0.0018	0.9248	0.0847	**0.0028**	0.0659	**0.0076**	0.0657	**0.0002**	0.0770	**0.0024**	0.0153	0.1214	0.0115	0.1740	0.0097	0.2098	0.0084	0.2080	0.0131	0.0508
45	0.0019	0.3418	0.0150	0.1310	0.0306	0.0536	0.0396	**0.0020**	0.0284	**0.0494**	−0.0021	0.6924	0.0026	0.3608	−0.0015	0.6160	0.0149	0.1206	−0.0020	1.0000
48	0.0002	0.4380	0.0388	**0.0268**	0.0261	0.1086	0.0065	0.2020	0.1331	**0.0000**	−0.0037	1.0000	0.0002	0.4436	−0.0038	1.0000	−0.0020	0.7064	−0.0011	0.6488
56	0.0083	0.0916	0.0457	**0.0154**	−0.0047	0.7696	0.0132	0.0630	0.0013	0.4400	0.0087	0.1722	−0.0020	0.6162	−0.0038	1.0000	−0.0037	0.9000	0.0203	**0.0160**
58	0.0005	0.4720	0.1137	**0.0006**	0.2011	**0.0000**	0.1082	**0.0000**	0.1312	**0.0000**	0.0292	**0.0288**	0.0010	0.4444	0.0306	**0.0252**	0.0212	0.0512	0.0545	**0.0000**
64	0.0257	**0.0040**	0.0618	**0.0066**	0.1319	**0.0004**	0.1099	**0.0000**	0.0750	**0.0008**	0.0158	0.1018	0.0519	**0.0060**	0.0121	0.1158	0.0117	0.1696	0.0162	**0.0340**
67	0.0008	0.3580	0.0136	0.1366	0.1220	**0.0000**	0.0349	**0.0070**	0.0291	0.0536	−0.0032	0.7948	0.0313	**0.0344**	0.0119	0.1472	0.0372	**0.0190**	−0.0018	0.8472
70	−0.0011	0.6616	−0.0046	0.8822	−0.0029	0.5428	0.0094	0.1124	0.0234	0.0882	0.0193	0.0882	0.0169	0.0794	−0.0029	0.7974	−0.0014	0.6210	0.0029	0.2818
77	0.0042	0.1720	0.0212	0.0966	0.0534	**0.0152**	0.0024	0.3260	0.0121	0.1564	−0.0029	0.7134	0.0088	0.2172	0.0007	0.4736	0.0007	0.3898	0.0120	0.0536
81	0.0020	0.3250	0.3542	**0.0000**	0.2493	**0.0000**	0.2925	**0.0000**	0.2972	**0.0000**	−0.0035	0.8668	−0.0038	1.0000	0.0487	**0.0090**	0.0598	**0.0036**	0.0057	0.1342
83	−0.0016	0.7902	0.0221	0.1042	0.0408	**0.0284**	0.0034	0.2860	0.0404	**0.0186**	0.0365	**0.0158**	0.0067	0.2568	0.0025	0.3068	0.0030	0.3218	0.0025	0.2768
84	0.0044	0.1534	0.0944	**0.0006**	0.1156	**0.0000**	0.1323	**0.0000**	0.1773	**0.0000**	0.0175	0.0744	0.0336	**0.0164**	0.0923	**0.0004**	0.0299	**0.0304**	−0.0019	0.9082
88	0.0082	0.1016	0.0431	**0.0228**	0.0566	**0.0134**	−0.0024	0.9250	0.0197	0.0868	−0.0012	0.5350	0.0012	0.3910	0.0162	0.0882	−0.0033	0.8034	0.0083	0.1210
92	0.0057	0.1600	−0.0047	1.0000	0.0033	0.3794	0.0065	0.1672	−0.0039	0.7828	−0.0031	0.8050	0.0026	0.3912	−0.0007	0.4626	0.0075	0.2160	−0.0013	0.6594
93	−0.0013	0.7280	0.0220	0.0874	0.0332	0.0524	0.0182	**0.0376**	−0.0038	0.7874	0.0079	0.2156	−0.0037	0.8982	0.0370	**0.0254**	−0.0022	0.7016	0.0057	0.1838
97	0.0026	0.2434	0.0388	**0.0350**	0.0996	**0.0020**	0.0268	**0.0140**	0.0449	**0.0116**	−0.0037	1.0000	0.0028	0.3096	−0.0025	0.6282	−0.0029	0.6990	−0.0016	0.7940
99	0.0231	**0.0122**	0.1549	**0.0000**	0.1208	**0.0004**	0.1717	**0.0000**	0.1846	**0.0000**	0.0128	0.1556	0.0246	0.0560	0.1321	**0.0000**	0.1446	**0.0000**	0.0057	0.1552
101	0.0045	0.1712	−0.0034	0.6846	0.0009	0.3838	−0.0023	0.9236	0.0243	0.0674	−0.0037	1.0000	−0.0038	1.0000	0.0189	0.0846	0.0008	0.4670	−0.0015	0.7212
111	0.0140	**0.0434**	0.3298	**0.0000**	0.4050	**0.0000**	0.3872	**0.0000**	0.3683	**0.0000**	0.0027	0.4348	−0.0001	0.4514	0.1011	**0.0000**	0.0804	**0.0012**	−0.0003	0.4832
114	0.0009	0.3592	0.0528	**0.0084**	−0.0053	0.9572	−0.0008	0.6002	0.0010	0.4122	−0.0013	0.5178	0.0007	0.4296	0.0064	0.2374	0.0208	0.0734	0.0111	0.0602
118	0.0086	0.0782	0.5122	**0.0000**	0.5123	**0.0000**	0.3814	**0.0000**	0.4593	**0.0000**	−0.0015	0.6448	0.0035	0.3720	0.3038	**0.0000**	0.2055	**0.0000**	−0.0018	0.8758
122	−0.0015	0.8532	0.0332	**0.0328**	0.0034	0.3476	0.0929	**0.0000**	0.0228	0.0620	−0.0026	0.6714	0.0267	**0.0490**	0.0168	0.0828	0.0158	0.0820	0.0140	**0.0446**
124	0.0002	0.4946	0.0030	0.3444	0.0138	0.1328	0.0029	0.2624	−0.0022	0.5980	−0.0024	0.6210	0.0082	0.2192	0.0066	0.2058	−0.0036	0.9052	0.0095	0.0798
125	0.0042	0.2190	0.0019	0.4136	0.0038	0.3780	0.0298	**0.0106**	0.0325	**0.0344**	−0.0004	0.5494	−0.0007	0.5500	0.0196	0.0774	0.0004	0.4562	−0.0005	0.5236
128	−0.0014	0.7126	0.0123	0.1582	0.0391	**0.0388**	0.0286	**0.0120**	0.0061	0.2790	−0.0008	0.5262	0.0036	0.3062	0.0065	0.2494	0.0017	0.4500	−0.0012	0.7122
131	0.0031	0.2536	0.0676	**0.0084**	0.0056	0.3538	0.1312	**0.0000**	0.0865	**0.0010**	0.0050	0.3104	0.0678	**0.0012**	0.0640	**0.0026**	0.0377	**0.0138**	−0.0009	0.6208
133	0.0002	0.4260	0.0884	**0.0014**	0.0800	**0.0058**	0.0512	**0.0010**	0.0730	**0.0018**	−0.0019	0.6252	0.0006	0.4652	−0.0034	0.7948	0.0019	0.3968	0.0047	0.2032
136	0.0014	0.3440	−0.0043	0.7900	0.0441	**0.0234**	N	N	0.0005	0.4324	−0.0025	0.7126	−0.0036	0.8154	−0.0024	0.6190	−0.0009	0.5486	0.0080	0.1152

N, No result was obtained for the comparison between Chinese Xibe group and Hungarian group at HLD6 and HLD136 loci since the genotyping results at these 2 loci were not included in the published data due to the detection of inconclusive genotypes.

*p*-values below 0.05 were in bold.
